# Impact of *TNF* -308 G>A (rs1800629) gene polymorphism in modulation of leprosy risk: a reappraise meta-analysis of 14 case–control studies

**DOI:** 10.1042/BSR20170806

**Published:** 2017-10-27

**Authors:** Mohammed Y. Areeshi, Raju K. Mandal, Sajad A. Dar, Arshad Jawed, Mohd Wahid, Mohtashim Lohani, Aditya K. Panda, Bhartendu N. Mishra, Naseem Akhter, Shafiul Haque

**Affiliations:** 1 Research and Scientific Studies Unit, College of Nursing and Allied Health Sciences, Jazan University, Jazan 45142, Saudi Arabia; 2 The University College of Medical Sciences and GTB Hospital, University of Delhi, Delhi 110095, India; 3 Department of Biosciences, Integral University, Lucknow 226026, Uttar Pradesh, India; 4 Centre for Life Sciences, Central University of Jharkhand, Ranchi 835205, Jharkhand, India; 5 Department of Biotechnology, Institute of Engineering and Technology, Lucknow 226021, Uttar Pradesh, India; 6 Department of Laboratory Medicine, Faculty of Applied Medical Sciences, Albaha University, Albaha 65431, Saudi Arabia

**Keywords:** cytokine, genetic model, leprosy, Meta-analysis, polymorphism, TNF

## Abstract

Purpose: Earlier studies have shown that tumor necrosis factor (*TNF*) -308 G>A (rs1800629) gene polymorphism is implicated in the susceptibility to leprosy, but results were inconsistent.

Methods: A meta-analysis of 14 studies involving 3327 leprosy cases and 3203 controls was performed to appraise the association of *TNF* -308 G>A polymorphism with leprosy using MEDLINE (PUBMED), EMBASE, and Google Scholar web databases.

Results: Overall, no significant association was observed in allelic (A vs. G: *P*=0.068; OR = 0.836, 95% CI = 0.689–1.013), homozygous (AA vs. GG: *P*=0.394; OR = 0.810, 95% CI = 0.499–1.315), heterozygous (GA vs. GG: *P*=0.059; OR = 0.780, 95% CI = 0.603–1.010), dominant (AA + GA vs. GG: *P*=0.067; OR = 0.797, 95% CI = 0.625–1.016), and recessive (AA vs. GG + GA: *P*=0.594; OR = 0.877, 95% CI = 0.542– 1.420) genetic models. Subgroup analysis showed no association in Asians. Whereas, reduced risk was found in allelic contrast (A vs. G: *P*=0.014; OR = 0.832, 95% CI = 0.718–0.963) and dominant models (AA + GA vs. GG: *P*=0.004; OR = 0.790, 95% CI = 0.673–0.928) of the mixed population.

Conclusions: *TNF* -308 G>A polymorphism is not associated with leprosy risk in the overall population. However, subgroup analysis demonstrated protective effect of the said polymorphism in leprosy risk in the Latin American population, but showed no association in the Asians.

## Introduction

Leprosy or Hansen’s disease is a chronic infection caused by the intracellular pathogen *Mycobacterium leprae* [1]. Leprosy remains a global public health concern and one of the most important preventable infectious disabilities in many developing countries [2]. In general, leprosy affects the skin and peripheral nerves and can cause irreversible impairment of nerve functions and consequent chronic disabilities. There are clinical and epidemiological evidence available that reveal leprosy doesn’t occur in most of the exposed individuals, this fact can be partly subjected to their genetic background and/or implication of the involvement of immune response genes [3]. In view of the spectrum of leprosy disease, several immune-response related genes have been implicated in the susceptibility and the severity of this dreadful disease. Also, earlier studies have proved that cytokine related genes play an important role in host–pathogen interaction [4]. Hence, elucidation of cytokine genetic determinants of host susceptibility to leprosy might facilitate the development of better preventive and therapeutic strategies.

*Tumor necrosis factor* (*TNF*) [previously known as *TNF-α* (*TNF-α*) or *TNFA*] is a pleiotropic cytokine that produced as a part of the host defense against the infection. *TNF* gene comprises of four exons with three intervening introns map between the *major histocompatibility complex* (*MHC*) class I and II regions on the short arm of chromosome 6. *TNF* plays a significant role in the control of mycobacterial growth and spread through the activation of macrophages, granulomas formation, and in the orchestration of the cellular immune response [5]. Thus, it is possible that the clinical outcomes of leprosy are affected by the propensity of the host to produce *TNF* in response to the infection of *M. leprae.* Also, a previous study of *TNF* knockout mice showed that *TNF* is indispensable for the resistance in infectious disease [6]. A number of single nucleotide polymorphisms have been discovered in the *TNF-α* locus and have shown to influence the rate of transcription and protein production of *TNF* and their association with various infectious diseases [7]. The locus -308 present in the promoter region of *TNF* gene has been much more considered than any other loci (i.e. -238 and -863) in correlation with the outcomes of infectious disease manifestation [7]. The more common *TNF1* allele has a guanine (G) residue, whereas the less common *TNF2* allele has an adenine (A) at position -308. *In vitro* studies with human peripheral blood monocytes have deciphered that the *TNF2* allele, whether homozygous or heterozygous, is linked with a higher production of *TNF* [8].

After knowing the clinical significance of *TNF* in the severity of clinical manifestation of various infectious diseases, including leprosy, it is important to explore its precise role in the development of leprosy. To date, numerous case–control studies have been done to appraise the relationship between the *TNF* -308 G>A gene polymorphism and risk of leprosy susceptibility, but results from those studies yielded inconsistent and conflicting outcomes. Still, it is unclear whether this polymorphism is associated with increased or decreased susceptibility to leprosy infection [9–22].

A recent study of Oliveira et al. [9] also reported varying results in contrast with the previously published meta-analysis of Cardoso et al. [15], where they failed to provide significant evidence for allelic association between the rs1800629 and leprosy risk [9]. Thus, Oliveira et al. [9] warranted the need of meta-analysis update with larger studies showing more accurate clinical phenotyping of leprosy subgroups for suitable power of the pooled study and to determine the potential role of *TNF* -308 (rs1800629) polymorphism as a genetic risk factor for leprosy.

Generally, the inconsistency in the results across many of the studies could possibly be related to the ethnicity of the population, sample size, and individual studies that have low power to evaluate the overall effect. Hence, in the light of above-mentioned contradictory findings from other researchers and their recommendations [9,15], and need of precise conclusion about this association, we performed this meta-analysis from the published literature of available case–control studies to clarify the role of -308 G>A polymorphism of *TNF* gene and leprosy risk. Meta-analysis is a statistical tool that is mainly used to explore the risk factors associated with the genetic diseases, as it employs a quantitative method to combine the data drawn from individual studies, where the sample sizes are too small to provide reliable conclusions.

## Materials and methods

### Strategy for literature search

We performed a PubMed (Medline), Google Scholar, and EMBASE online web database search covering all research studies published with a combination of the following key words: i.e. tumor necrosis factor OR tumor necrosis factor-alpha OR *TNFA* OR *TNF-α* OR *TNF* gene (polymorphism OR variant OR mutation) AND leprosy susceptibility OR risk (last updated on December, 2016). We examined potentially relevant genetic association studies by inspecting their titles and abstracts, and obtained the most pertinent publication matching with the above said preset eligibility criteria for a closer examination. In addition to the online database search, the references given in the retrieved research articles were also screened for other potentially relevant articles that may have been overlooked in the preliminary search.

### Inclusion and exclusion criteria

In order to reduce heterogeneity and facilitate the apt interpretation of the present study, the published reports included in the present meta-analysis had to meet all the below given criteria: i.e. (a) they must have done case–control studies between *TNF*-308 G>A gene polymorphism and leprosy risk; (b) clearly described confirmed leprosy patients and leprosy disease free controls; (c) have available genotype frequency in both the cases and the controls; (d) published in the English language; (e) data collection and analysis methodology must be statistically acceptable. In addition to above, when the case–control study was included in more than one research article using the same set of case series, we selected the research study that incorporated the largest number of the individuals. The major reasons for study exclusion were: (a) duplicate or overlapping publication, (b) study design based on only leprosy cases, (c) genotype frequency not reported, and (d) the data of review or abstract.

### Data extraction

For each retrieved study, the procedural quality assessment and data extraction were independently summarized in duplicate copies by the two independent investigators (SAD & RKM) following a standard protocol. During the data extraction process, data-collection form was used to ensure the accuracy of the collected data by stringently following the preset inclusion/exclusion criteria as mentioned above, and sequential exclusion of the unsuitable studies. In case of disagreement between the above-mentioned two investigators on any item related with the data collected from the selected studies, the issue was fully debated and deliberated with the investigators to attain a final consensus. Also, in case failure of reaching consensus between the two investigators, an agreement was achieved following an open discussion with the adjudicator (SH). The main characteristics abstracted from the retrieved publications comprise the name of the first author, the country of origin, publication year, number of cases and controls, source of cases and controls, study type, genotype frequencies, and association with leprosy.

### Quality assessment of the included studies

Methodological quality evaluation of the selected studies was performed independently by two investigators (RKM & SAD) by following the Newcastle–Ottawa Scale (NOS) of quality assessment [23]. The NOS quality assessment criteria included three major aspects: (i) subject selection: 0–4 points, (ii) comparability of subject: 0–2 points, and (iii) clinical outcome: 0–3 points. Selected case–control studies that were gained five or more stars can be considered as of moderate to good quality [24].

### Statistical analysis

In order to appraise the association between the *TNF* -308 G>A gene polymorphism and susceptibility to leprosy risk, pooled ORs and their corresponding 95% CIs were estimated. Heterogeneity assumption was determined by the chi-square-based *Q*-test [25]. Heterogeneity was considered significant at *P*-value < 0.05. The collected data from single comparison was combined using a fixed effects model [26], when no heterogeneity was present. Or else, the random-effects model [27] was employed for the pooling of the data. Moreover, *I*^2^ statistics was used to quantify the interstudy variability and larger values indicated an increasing degree of heterogeneity [28]. Hardy–Weinberg equilibrium (HWE) in the controls was estimated by the chi-square test. Funnel plot asymmetry was measured by Egger’s regression test, which is a linear regression approach of measuring the funnel plot asymmetry on the natural logarithm scale of the OR. The significance of the intercept was measured by the *t*-test (*P*-value < 0.05 was considered as a representation of statistically significant publication bias). Also, ethnicity was adopted to perform the subgroup stratified analysis, when data were available. A comparative assessment of ‘meta-analysis’ software programs was done by using the link: http://www.meta-analysis.com/pages/comparisons.html, and finally the Comprehensive Meta-Analysis (CMA) software program Version 2., from Biostat (NJ), U.S.A. was used to perform all the statistical investigations involved in this pooled analysis.

## Results

### Characteristics of the published studies included in this meta-analysis

**Figure 1:**
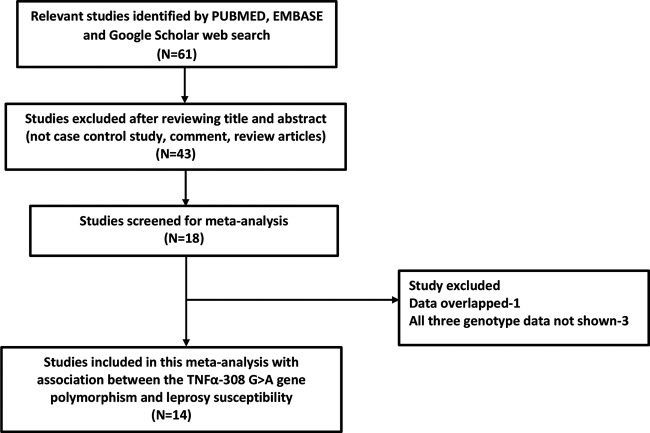
Flow Diagram Identification and selection of pertinent studies for this meta-analysis.

A total of 14 articles were finally selected after systematic literature search from PubMed (Medline), EMBASE, and Google Scholar online web-based databases. All the retrieved publications were scrutinized carefully by reading their titles and abstracts, and the full-texts for the potentially relevant publications were further checked for their suitability for this meta-analysis (Figure 1). Research publications either showing *TNFα* gene polymorphism to predict survival in leprosy patients or considering leprosy variants as indicators for response to therapy were excluded straightaway. Likewise, research studies evaluating the levels of *TNF* mRNA or protein expression or germane review articles were also disqualified from this meta-analysis. For this pooled study, only case–control or cohort design studies were included stating the frequency of all the three genotypes. In addition to the online web database search, the supporting references listed in the retrieved articles were also reviewed for other potential case–control studies. After cautious screening and following the stringent inclusion and exclusion criteria, 14 eligible original publications were finally considered for this meta-analysis (Table 1). The distribution of genotypes, HWE *P*-values in the controls, and susceptibility toward leprosy risk have been given in Table 2. All the selected studies (14 in number) were examined for the overall quality following the NOS and most of the studies (>80%) scored five stars or more, indicating a modest to decent quality (Table 3).

**Table 1:** Main characteristics of all studies included in the meta-analysis

First author, Year [Ref. No.]	Country	Ethnicity	Controls	Cases	Study	Association observed
Oliveira et al., 2016 [9]	Brazil	Latin American	331	326	HB	No risk
Sykam et al., 2015 [10]	India	Asian	129	88	HB	No risk
Tarique et al., 2015 [11]	India	Asian	120	102	HB	GA genotype shown reduced risk
Silva et al., 2015 [12]	Brazil	Latin American	253	108	HB	AT genotype decreased risk
Felix et al., 2012 [13]	Mexico	Latin American	144	68	HB	No risk
Lima et al., 2012 [14]	Brazil	Latin American	68	46	PB	No risk
Cardoso et al., 2011 [15]	Brazil	Latin American	1036	1146	PB	A allele shown protective risk
Velayati et al., 2011 [16]	Iran	Asian	72	3	HB	No risk
Sapkota et al., 2010 [17]	Nepal	Asian	94	820	HB	AA genotype shown protective
Settin et al., 2007 [18]	Egypt	African	98	47	HB	GG shown risk
Vejbaesya et al., 2007 [19]	Thailand	Asian	140	37	HB	GA genotype shown risk
Fitness et al., 2004 [20]	Malawi	African	258	216	PB	No risk
Santos et al., 2002 [21]	Brazil	Latin American	300	92	HB	No risk
Roy et al., 1997 [22]	India	Asian	160	228	PB	GA genotype shown risk in different types of leprosy

Abbreviations: HP, hospital based; PB, patient based.

**Table 2:** Genotypic distribution of *TNF* -308 G>A (rs1800629) gene polymorphism studies included in the meta-analysis

Authors and year	Controls				Cases				HWE	Power value	
	Genotype			Minor allele	Genotype			Minor allele			
	GG	GA	AA	MAF	GG	GA	AA	MAF	*P*-value	ES (0.2)	ES (0.1)
Oliveira et al., 2016	270	57	4	0.098	258	65	3	0.108	0.35	0.988	0.471
Sykam et al., 2015	115	13	1	0.058	81	6	1	0.045	0.08	0.605	0.167
Tarique et al., 2015	84	34	2	0.158	87	12	3	0.088	0.01	0.616	0.170
Silva et al., 2015	215	36	2	0.079	95	11	2	0.069	0.26	0.851	0.264
Felix et al., 2012	123	20	1	0.076	60	8	0	0.058	0.11	0.593	0.164
Lima et al., 2012	55	13	0	0.095	35	10	1	0.130	0.04	0.331	0.106
Cardoso et al., 2011	791	230	15	0.125	930	200	16	0.101	0.01	1.000	0.968
Velayati et al., 2011	57	15	0	0.104	2	1	0	0.166	0.28	0.222	0.085
Sapkota et al., 2010	79	13	2	0.090	743	74	3	0.048	0.13	0.999	0.631
Settin et al., 2007	6	81	11	0.525	8	37	1	0.423	0.27	0.418	0.123
Vejbaesya et al., 2007	127	13	0	0.046	29	8	0	0.108	0.03	0.505	0.142
Fitness et al., 2004	201	51	6	0.122	173	42	1	0.101	0.04	0.940	0.344
Santos et al., 2002	59	30	0	0.168	243	49	8	0.108	0.83	0.883	0.286
Roy et al., 1997	151	9	0	0.028	208	17	3	0.050	0.98	0.879	0.283

Abbreviations: HWE, Hardy–Weinberg equilibrium; MAF, minor allele frequency.

**Table 3:** Quality assessment conducted according to the NOS for all the studies included in the meta-analysis

First author and year	Quality indicators		
	Selection	Comparability	Exposure
Oliveira et al., 2016	****	*	**
Sykam et al., 2015	***	*	***
Tarique et al., 2015	***	*	**
Silva et al., 2015	***	*	***
Felix et al., 2012	****	*	***
Lima et al., 2012	***	*	**
Cardoso et al., 2011	***	*	**
Velayati et al., 2011	***	*	***
Sapkota et al., 2010	****	*	**
Settin et al., 2007	***	*	*
Vejbaesya et al., 2007	***	*	**
Fitness et al., 2004	****	*	***
Santos et al., 2002	***	*	**
Roy et al., 1997	***	*	**

### Diagnosis of publication bias

**Figure 2:**
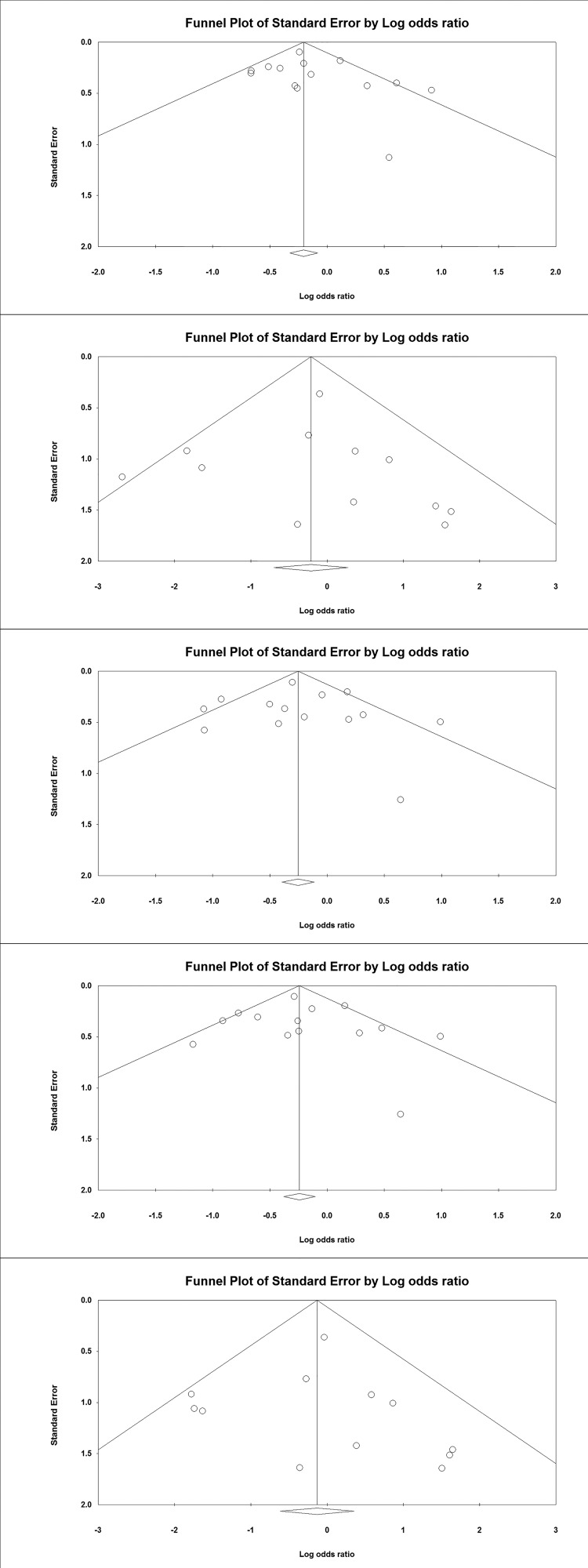
Funnel Plots: Assessment of publication bias shown with Funnel plots in studies assaying odds of leprosy associated with the *TNF* -308 G>A gene polymorphism for overall analysis (odds ratio against standard error in different genetic models).

Begg’s funnel plot and Egger’s test were done to observe the publication bias among the selected case–control studies for the present meta-analysis. As depicted in Table 4, no publication bias was found among all the comparison models using both Egger’s and Begg’s regression analysis in all the genetic models and the allelic contrast (Figure 2).

**Table 4:** Statistics to test publication bias and heterogeneity in meta-analysis: overall analysis

Comparisons	Egger’s regression analysis			Heterogeneity analysis			Model used for this meta-analysis
	Intercept	95% confidence interval	*P*-value	*Q*-value	*P*_heterogeneity_	*I*^2^ (%)	
A vs. G	0.64	−0.84 to 2.14	0.36	22.53	0.04	42.31	Random
AA vs. GG	0.14	−1.46 to 1.76	0.84	14.84	0.19	25.88	Fixed
GA vs. GG	0.17	−1.51 to 1.85	0.82	28.92	0.007	55.06	Random
AA + GA vs. GG	0.27	−1.35 to 1.91	0.71	27.31	0.01	52.39	Random
AA vs. GG+GA	0.26	−1.25 to 1.77	0.70	13.04	0.29	15.66	Fixed

### Test of heterogeneity

Heterogeneity among the studies included in the present meta-analysis was evaluated using the chi-squared-based *Q*-test and *I*^2^ statistics. Substantial heterogeneity was found in three genetic models (i.e. A vs. G, GA vs. GG, AA + GA vs. GG). Thus, random and fixed effects models were applied to synthesize the data (Table 4).

### Sensitivity analysis

**Figure 3:**
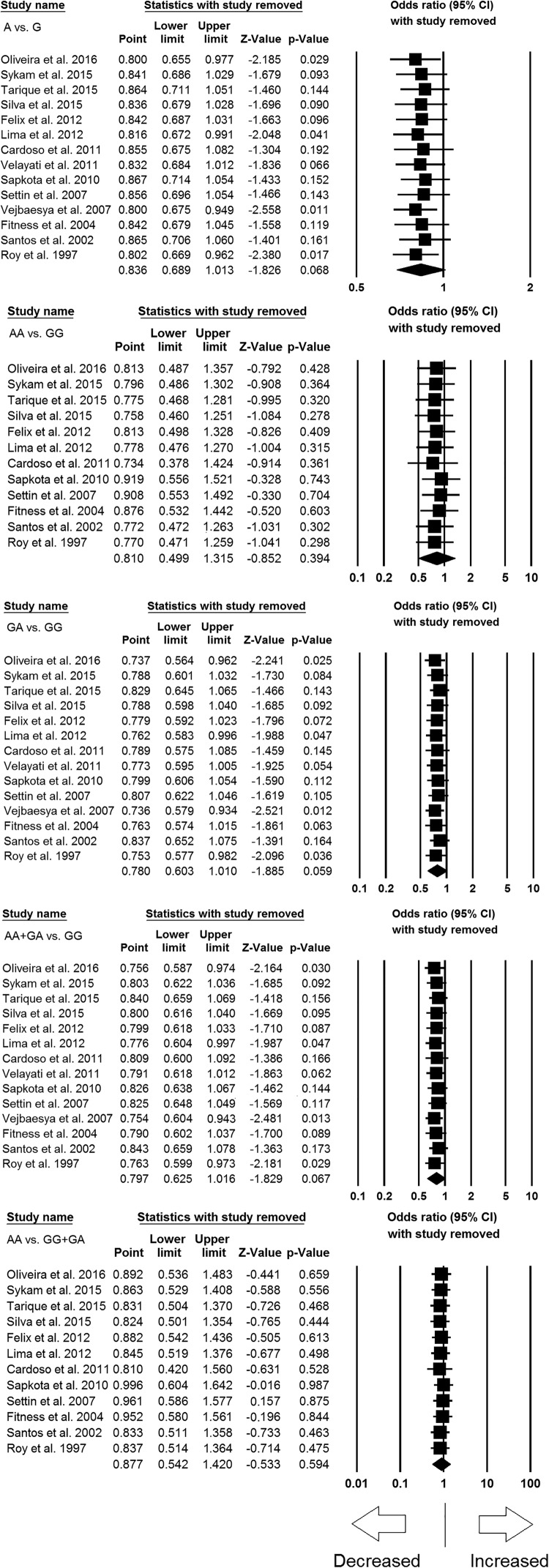
Sensitivity analysis to evaluate the influence of each individual study on the pooled OR by deleting one single study each time for overall analysis (for all the genetic models). Black squares represent the value of OR and the size of the square indicates the inverse proportion relative to its variance. Horizontal line is the 95% CI of OR.

Sensitivity analysis was carried out to evaluate the impact of each individual study on the pooled ORs by removing one single study each time. The outcomes revealed that no individual study influenced the pooled OR significantly, and indicated the stability of the current meta-analysis (Figure 3).

### Quantitative synthesis

**Figure 4:**
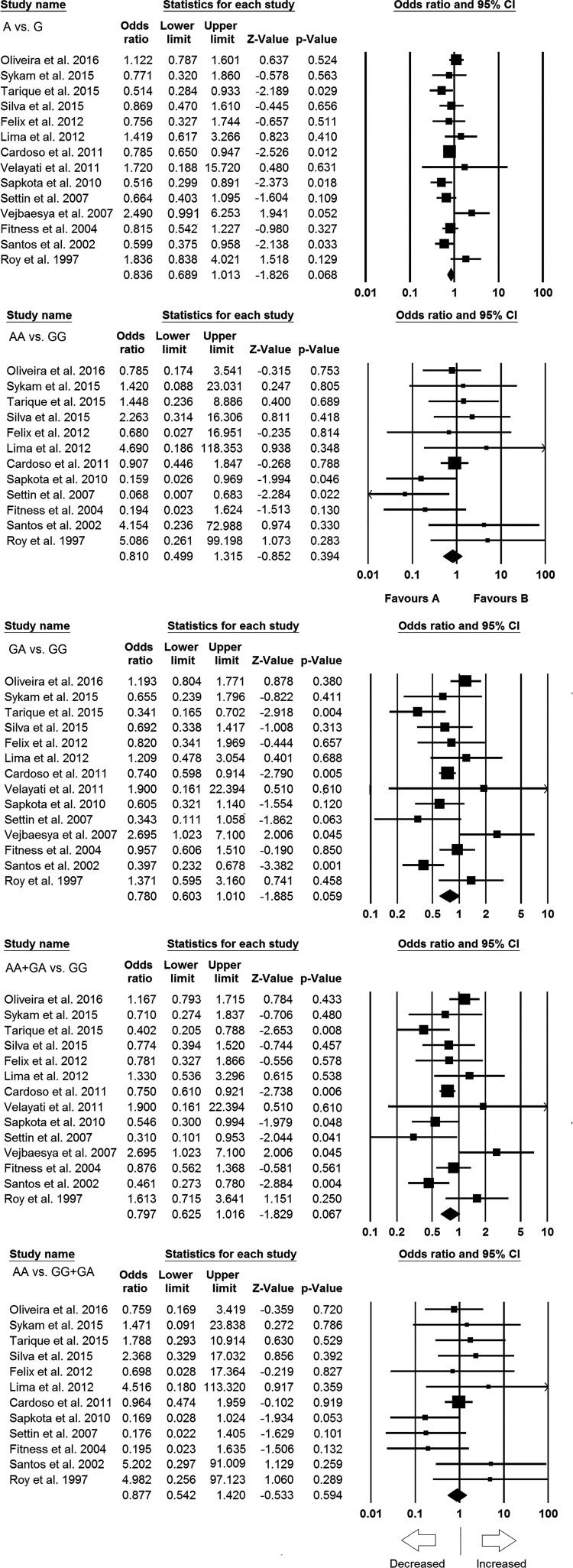
Forest plot of OR with 95% CI of leprosy risk associated with the *TNF* -308 G>A gene polymorphism for overall population. Black squares represent the value of OR and the size of the square indicates the inverse proportion relative to its variance. Horizontal line is the 95% CI of OR.

We pooled all the 14 studies together that resulted into 3327 confirmed leprosy cases and 3203 healthy controls for the assessment of overall association between the *TNF* -308 G>A polymorphism and risk of leprosy infection. The pooled ORs from the overall studies suggested no association with increased or decreased risk between *TNF* -308 G>A gene polymorphism and leprosy risk in allelic contrast (A vs. G: *P*=0.068; OR = 0.836, 95% CI = 0.689–1.013), homozygous (AA vs. GG: *P*=0.394; OR = 0.810, 95% CI = 0.499–1.315), heterozygous (GA vs. GG: *P*=0.059; OR = 0.780, 95% CI = 0.603–1.010), dominant (AA + GA vs. GG: *P*=0.067; OR = 0.797, 95% CI = 0.625–1.016), and recessive (AA vs. GG + GA: *P*=0.594; OR = 0.877, 95% CI = 0.542–1.420) genetic models (Figure 4; Table 4).

### Subgroup analysis: association of the *TNF* -308 G>A polymorphism and risk of leprosy infection in Asian and Latin American population

A stratified subgroup analysis based on the ethnicity of the enrolled subjects was performed to explore the effect of ethnicity (Asian and Latin American) on the relationship between *TNF* -308 G>A gene polymorphism and the risk of leprosy development.

### Subgroup analysis of Asian population

**Figure 5:**
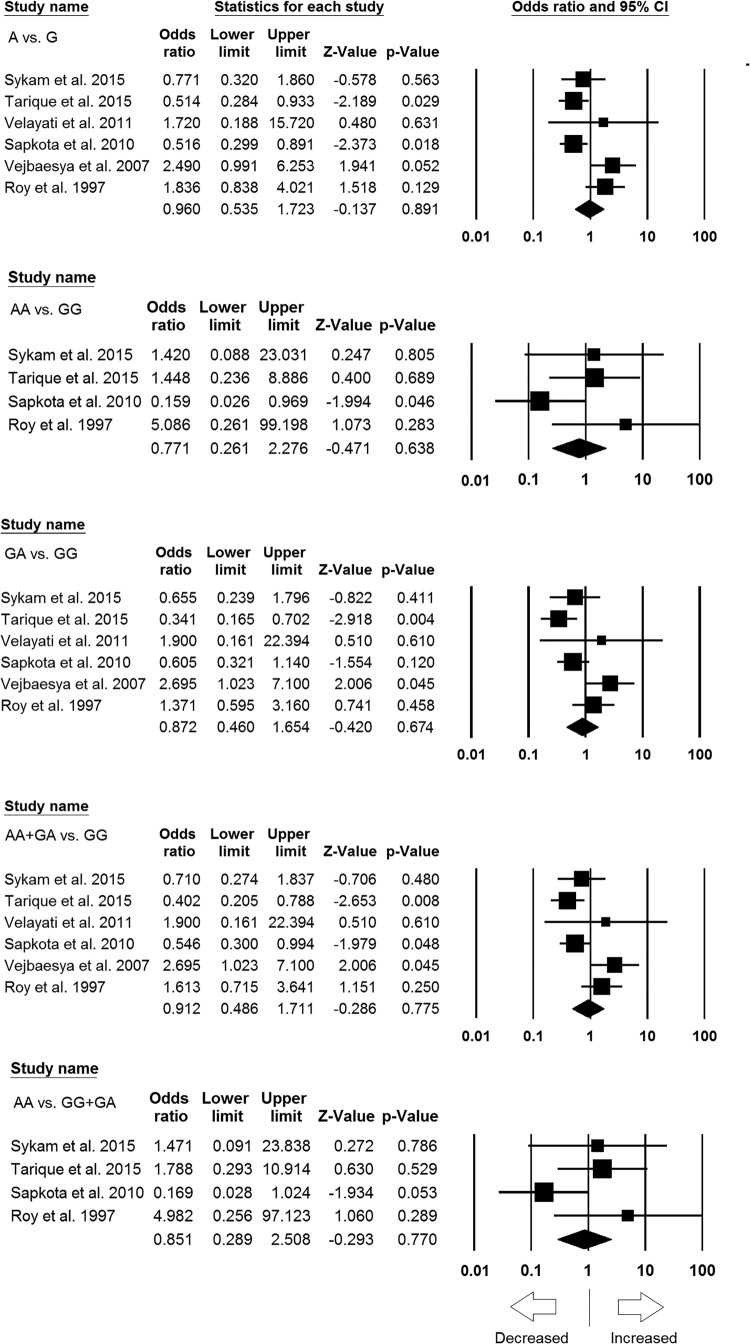
Forest plots of ORs with 95% CI of leprosy risk associated with the *TNF* -308 G>A gene polymorphism in Asian population. Black squares represent the value of OR and the size of the square indicates the inverse proportion relative to its variance. Horizontal line is the 95% CI of OR.

Six case–control studies resulted 715 controls and 1278 cases were included for subgroup analysis of Asian (India, Thailand, Iran, and Nepal) population. During the analysis, no publication bias was detected; whereas, significant heterogeneity was observed in three genetic models (Table 5) (Supplementary Figures S1 and S2). We conducted analyses using random and fixed models and observed no significant association of leprosy susceptibility in all genetic models, i.e. allele model (A vs. G: *P*=0.891; OR = 0.960, 95% CI = 0.535–1.723), homozygous model (AA vs. GG: *P*=0.638; OR = 0.771, 95% CI = 0.261–2.276), heterozygous model (GA vs. GG: *P*=0.674; OR = 0.872, 95% CI = 0.460–1.654), dominant model (AA + GA vs. GG: *P*=0.775; OR = 0.912, 95% CI = 0.486–1.711), and recessive model (AA vs. GG + GA: *P*=0.770; OR = 0.851, 95% CI = 0.289–2.508) (Figure 5).

**Table 5:** Statistics to test publication bias and heterogeneity in this meta-analysis: Asian population

Comparisons	Egger’s regression analysis			Heterogeneity analysis			Model used for the meta-analysis
	Intercept	95% confidence interval	*P*-value	*Q*-value	*P*_heterogeneity_	*I*^2^ (%)	
A vs. G	2.83	−2.38 to 8.04	0.20	15.19	0.01	67.09	Random
AA vs. GG	3.24	−8.65 to 15.15	0.36	5.12	0.16	41.49	Fixed
GA vs. GG	2.21	−3.77 to 8.19	0.36	14.31	0.01	65.06	Random
AA + GA vs. GG	2.54	−3.06 to 8.16	0.27	14.97	0.01	66.60	Random
AA vs. GG + GA	2.99	−9.66 to 15.66	0.41	5.24	0.15	42.83	Fixed

### Subgroup analysis of Latin American population

**Figure 6:**
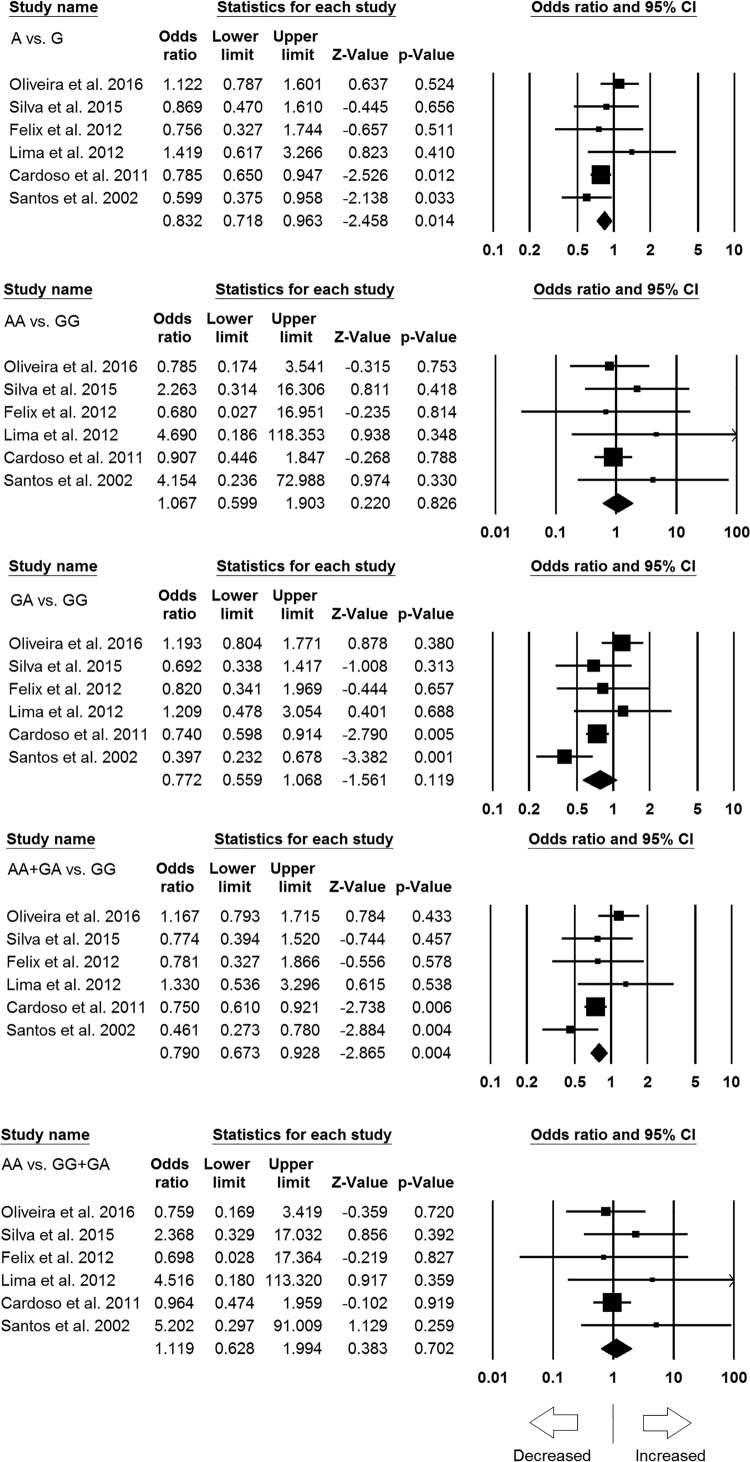
Forest plots of OR with 95% CI of leprosy risk associated with the *TNF* -308 G>A gene polymorphism in the mixed population. Black squares represent the value of OR and the size of the square indicates the inverse proportion relative to its variance. Horizontal line is the 95% CI of OR.

Similar to the subgroup analysis of Asian population, six studies resulted 2132 controls and 1786 cases were also included in the subgroup analysis of Latin American (i.e. Brazil and Mexico) population. During the analysis, no publication bias was observed but heterogeneity was found in one model (Table 6) (Supplementary Figures S3 and S4). Interestingly, we found protective association of leprosy risk with allelic contrast (A vs. G: *P*=0.014; OR = 0.832, 95% CI = 0.718–0.963) and dominant genetic model (AA + GA vs. GG: *P*=0.004; OR = 0.790, 95% CI = 0.673–0.928). Whereas, remaining three genetic models, i.e. homozygous (AA vs. GG: *P*=0.826; OR = 1.067, 95% CI = 0.599–1.903), heterozygous (GA vs. GG: *P*=0.119; OR = 0.772, 95% CI = 0.559–1.068), and recessive (AA vs. GG + GA: *P*=0.702; OR = 1.119, 95% CI = 0.628–1.994) genetic models showed no association with increased or decreased risk of leprosy (Figure 6).

**Table 6:** Statistics to test publication bias and heterogeneity in this meta-analysis: Latin American population

Comparisons	Egger’s regression analysis			Heterogeneity analysis			Model used for the meta-analysis
	Intercept	95% confidence interval	*P*-value	*Q*-value	*P*_heterogeneity_	*I*^2^ (%)	
A vs. G	0.53	−2.19 to 3.27	0.61	6.61	0.25	24.46	Fixed
AA vs. GG	0.80	−0.45 to 2.07	0.14	2.66	0.75	0.00	Fixed
GA vs. GG	0.11	−3.69 to 3.91	0.93	11.77	0.03	57.55	Random
AA + GA vs. GG	0.32	−3.05 to 3.70	0.80	9.46	0.09	47.17	Fixed
AA vs. GG + GA	0.80	−0.56 to 2.16	0.17	2.89	0.71	0.00	Fixed

## Discussion

The identification of host genes and genetic variations that are important in susceptibility and resistance to leprosy would assist in better understanding of the pathogenesis of leprosy and perhaps lead to new approaches for the diagnosis and treatment or prophylaxis. As we know that leprosy is one of the most common chronic infectious diseases and a well-established genetic marker assuredly would have a noteworthy influence on screening and prevention of leprosy. Indeed, genome-wide association studies have successfully described genetic risk factors involved in leprosy [29].

Cytokine polymorphism has been considered to be of playing significant role in host genetic factors of leprosy. Both, *in vitro* and *in vivo* studies have shown the presence of genetic variants within the coding or noncoding sequences of cytokine genes that can modify the efficiency of transcription of these genes, and consequently the production of cytokines. Significant role played by *TNF* in inflammation and its relevance to infectious disease has led to prodigious attention in both the regulation of the *TNF* gene, and the likelihood that polymorphisms of the gene or deregulation of its production may perhaps associated with pathology of leprosy. *TNF* also plays a potential role in the pathogenesis of acute inflammatory leprosy reactions liable for various outcomes that characterize leprosy. Earlier studies have shown that *TNF* production by the cells of -308 GG homozygous individuals and GA heterozygote individuals produced varying results [30], and reported higher *TNF* production by the cells from GA donors than by GG [30]. Unexpectedly, other research studies have stated no significant effect on *TNF* expression [31]. Despite many efforts, the molecular and biological mechanism of interaction between the *TNF* gene polymorphism and risk of leprosy yet elucidated precisely. Even to date, the results of candidate *TNF* (-308 G>A) gene based case–control studies are inconsistent in relation with leprosy risk. Some clinical case–control studies have found positive association while others have reported negative. Majorly, the results of the studies generated could be of inadequate statistical power due to individual studies with small sample sizes or variations that existed in different populations. Therefore, larger sample size with pooled analysis and subgroup analysis is required to evaluate the potential role of *TNF* -308 G>A polymorphism as a genetic risk factor for leprosy infection. Pooled ORs generated from large sample size and sufficient statistical power from different studies have the advantage of minimizing the random errors [32]. Circa, from the last 10–15 years, a meta-analysis has been well recognized as an efficient statistical tool to resolve a wide variety of clinical questions by pooling and reviewing the earlier published quantitative data. In this meta-analysis, we have included 14 eligible case–control studies comprising 3327 cases and 3203 healthy controls and analyzed the pooled ORs and *P*-value to appraise the precise association between the *TNF* -308 G>A polymorphism and leprosy risk. Most of the included studies scored five or more stars in NOS quality assessment and suggested good to moderate quality by clearly stating about the sample size, genotype, inclusion criteria of leprosy patients, and healthy controls. Interestingly, we found no association between the *TNF* -308 G>A polymorphism and leprosy susceptibility under any genetic models in overall population analysis. Based upon the findings, we can speculate that the numerous polymorphic sites (in the promoter and the coding regions of *TNF* gene) might serve to keep this gene under tight control and influence the expression of *TNF*. Thus, some haplotype combinations might be conserved in the certain population to protect against pathogens and others to control expression. Also, gene reporter assay study reported that A allele of -308 polymorphism does not influence *TNF* gene transcription [33]. Other studies also reported that -308 G>A polymorphism leads to different transcription rate in *TNF* production [34,35]. Recently, it has been reported that the minor A allele is related with low levels of *TNF* mRNA in peripheral blood total leukocytes of leprosy patients [9].

In the subgroup analysis, no significant association was observed in the Asian population. As the Asian subgroup is possibly diverse genetically, thus colony based analysis is needed for more precise conclusion. Whereas, protective association was observed in the Latin American population. These findings suggest that there could be an interaction of -308 G>A polymorphism and the level of *TNF* production, which might be protective against leprosy among Latin American population but not in Asian population. However, more studies of Mexican and Brazilian population should be taken into the meta-analysis to further confirm our results.

Previous meta-analysis by Cardoso et al. [15] also found protective association between *TNF* -308 G>A gene polymorphism and leprosy risk. As a limitation, the meta-analysis of Cardoso et al. included only seven studies and did not examine Asian population. In addition, the meta-analysis presented by Cardoso et al. [15], only evaluated Brazilian population in mixed ethnicity that is considered as population of different ethnicities. We have significantly improved the present meta-analysis by including 14 studies, which is almost double in number of studies mentioned in the earlier analysis along with subgroup analysis by Asian and mixed ethnicity.

Earlier findings suggest that susceptibility toward leprosy is polygenic in nature and possibly multiple candidate genes are involved in determining the resistance or susceptibility to leprosy. Hence, because of the multifactorial nature of leprosy infection and complex nature of the immune system, *TNF* -308 G>A genetic polymorphism cannot be solely responsible for the predisposition of leprosy and may this polymorphism interacts with other polymorphisms present in linkage disequilibrium of this gene to cause risk.

In addition to the above-mentioned improvements, there are certain limitations of the present study that needs to be addressed in future studies with larger sample size. First, significant heterogeneity was observed in some of the genetic models, when all the studies were included. In subgroup analysis, heterogeneity was much lower in the Latin American population suggesting that ethnic-specific genetic variation might be the key factor responsible for heterogeneity. Second, studies published in the English language and abstracted and indexed by the selected (PubMed-Medline, EMBASE and Google Scholar) electronic databases for the data analysis; it is possible that some relevant studies might publish in languages other than the English or indexed in other electronic databases, may have missed. Third, the abstracted data from the included studies were not stratified by severity of the leprosy infection, and the current results are based on unadjusted parameters. Fourth, authors fail to test the gene–environment interactions due of inadequate information available in the primary published studies included in the present analysis.

Despite above limitations, this pooled study has some advantages over previous studies. First, this meta-analysis included larger number of studies to enhance the statistical power of the study which provided enough powerful evidence to reach on precise and robust conclusion. Second, no publication bias was detected and further sensitivity analysis also supported the reliability of our results. Also, all the included studies were of good to modest quality fulfilling the preset needful criteria as tested by NOS quality assessment scale.

## Conclusions

In conclusion, this meta-analysis did not demonstrate powerful evidence to identify *TNF* -308 G>A gene polymorphism as a significant biomarker for leprosy susceptibility in the overall population. However, a significant protective association was observed in allele and dominant models of Latin American population, but not in Asian population. As *TNF* plays a significant role in immune response against *M. leprae*, further larger well-designed case–control studies are warranted to support and conclude our current findings. Overall, the present study would greatly aid for comprehensive understanding of the link between the *TNF* -308 G>A polymorphism and leprosy risk globally.

## Supporting information

## Abbreviations

CI: Confidence Interval
HWE: Hardy–Weinberg Equilibrium
MHC: Major Histocompatibility Complex
NOS: Newcastle–Ottawa Scale
OR: Odds Ratio
TNF: Tumor Necrosis Factor
