# Supplementary material for: Impact of TNF -308 G>A (rs1800629) gene polymorphism in modulation of leprosy risk: a reappraise meta-analysis of 14 case–control studies
Source: Biosci Rep. 2017 Oct 27;37(5):BSR20170806. doi: 10.1042/BSR20170806 (PMC5664359; doi:10.1042/BSR20170806)

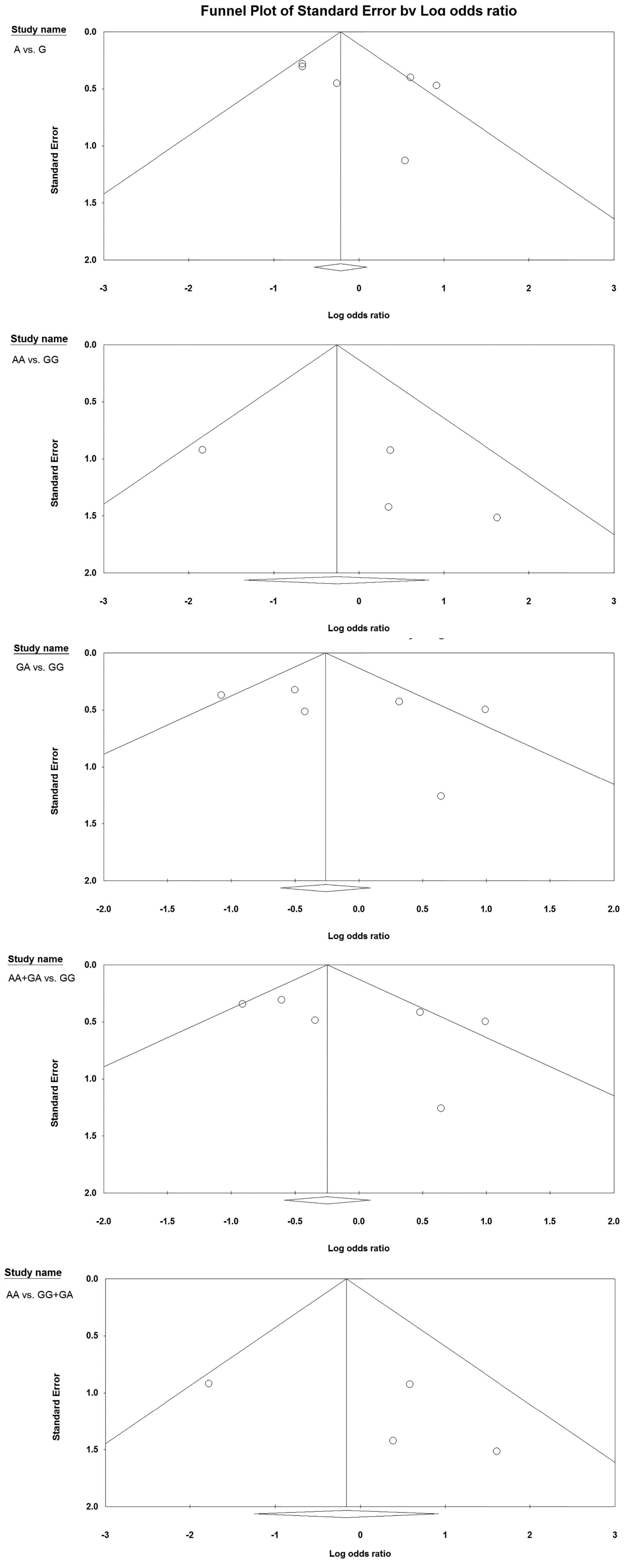

Funnel Plot of Standard Error by Log odds ratio
Study name
A vs. G
Standard Error
0.0
0.5
1.0
1.5
2.0
-3
-2
-1
0
1
2
3
Log odds ratio
Study name
AA vs. GG
Standard Error
0.0
0.5
1.0
1.5
2.0
-3
-2
-1
0
1
2
3
Log odds ratio
Study name
GA vs. GG
Standard Error
0.0
0.5
1.0
1.5
2.0
-2.0
-1.5
-1.0
-0.5
0.0
0.5
1.0
1.5
2.0
Log odds ratio
Study name
AA+GA vs. GG
Standard Error
0.0
0.5
1.0
1.5
2.0
-2.0
-1.5
-1.0
-0.5
0.0
0.5
1.0
1.5
2.0
Log odds ratio
Study name
AA vs. GG+GA
Standard Error
0.0
0.5
1.0
1.5
2.0
-3
-2
-1
0
1
2
3
Log odds ratio

Study name	Statistics with study removed				
A vs. G	Point	Lower limit	Upper limit	Z-Value	p-Value
Sykam et al. 2015	1.024	0.503	2.085	0.065	0.948
Tarique et al. 2015	1.146	0.569	2.307	0.381	0.703
Velayati et al. 2011	0.931	0.501	1.731	-0.226	0.821
Sapkota et al. 2010	1.150	0.577	2.292	0.396	0.692
Vejbaesya et al. 2007	0.775	0.456	1.314	-0.947	0.344
Roy et al. 1997	0.816	0.450	1.480	-0.669	0.504
	0.960	0.535	1.723	-0.137	0.891

Odds ratio (95% CI) with study removed

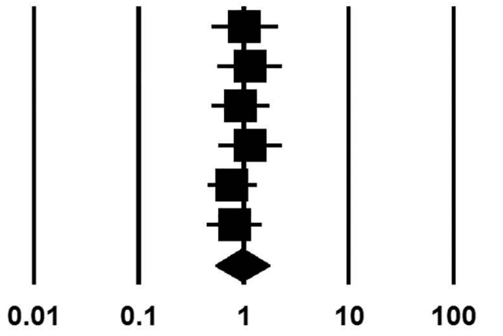

Study name	Statistics with study removed				
AA vs. GG	Point	Lower limit	Upper limit	Z-Value	p-Value
Sykam et al. 2015	0.692	0.214	2.239	-0.615	0.538
Tarique et al. 2015	0.544	0.141	2.096	-0.885	0.376
Sapkota et al. 2010	1.871	0.483	7.241	0.907	0.364
Roy et al. 1997	0.577	0.181	1.847	-0.926	0.355
	0.771	0.261	2.276	-0.471	0.638

Odds ratio (95% CI) with study removed

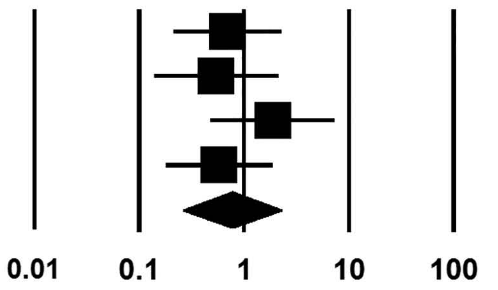

Study name	Statistics with study removed				
GA vs. GG	Point	Lower limit	Upper limit	Z-Value	p-Value
Sykam et al. 2015	0.939	0.433	2.033	-0.161	0.872
Tarique et al. 2015	1.088	0.588	2.016	0.269	0.788
Velayati et al. 2011	0.836	0.425	1.645	-0.519	0.604
Sapkota et al. 2010	0.985	0.426	2.280	-0.035	0.972
Vejbaesya et al. 2007	0.667	0.392	1.135	-1.493	0.136
Roy et al. 1997	0.791	0.377	1.661	-0.619	0.536
	0.872	0.460	1.654	-0.420	0.674

Odds ratio (95% CI) with study removed

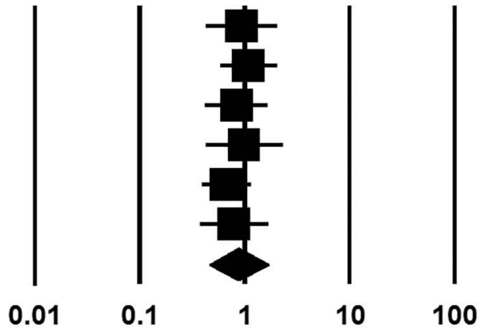

Study name	Statistics with study removed				
AA+GA vs. GG	Point	Lower limit	Upper limit	Z-Value	p-Value
Sykam et al. 2015	0.979	0.456	2.102	-0.054	0.957
Tarique et al. 2015	1.124	0.571	2.209	0.338	0.736
Velayati et al. 2011	0.878	0.452	1.707	-0.382	0.702
Sapkota et al. 2010	1.070	0.484	2.365	0.167	0.868
Vejbaesya et al. 2007	0.710	0.413	1.222	-1.236	0.217
Roy et al. 1997	0.797	0.401	1.584	-0.647	0.518
	0.912	0.486	1.711	-0.286	0.775

Odds ratio (95% CI) with study removed

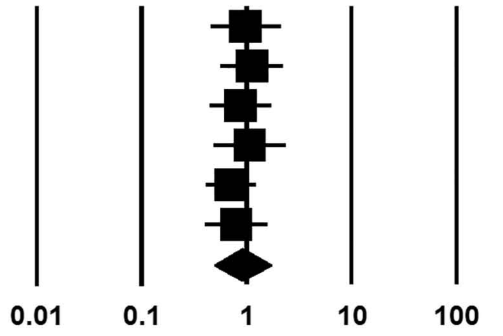

Study name	Statistics with study removed				
AA vs. GG+GA	Point	Lower limit	Upper limit	Z-Value	p-Value
Sykam et al. 2015	0.772	0.239	2.495	-0.432	0.666
Tarique et al. 2015	0.563	0.146	2.169	-0.834	0.404
Sapkota et al. 2010	2.111	0.547	8.152	1.084	0.278
Roy et al. 1997	0.650	0.204	2.073	-0.729	0.466
	0.851	0.289	2.508	-0.293	0.770

Odds ratio (95% CI) with study removed

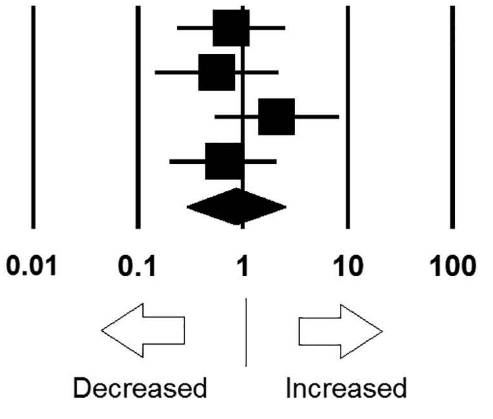

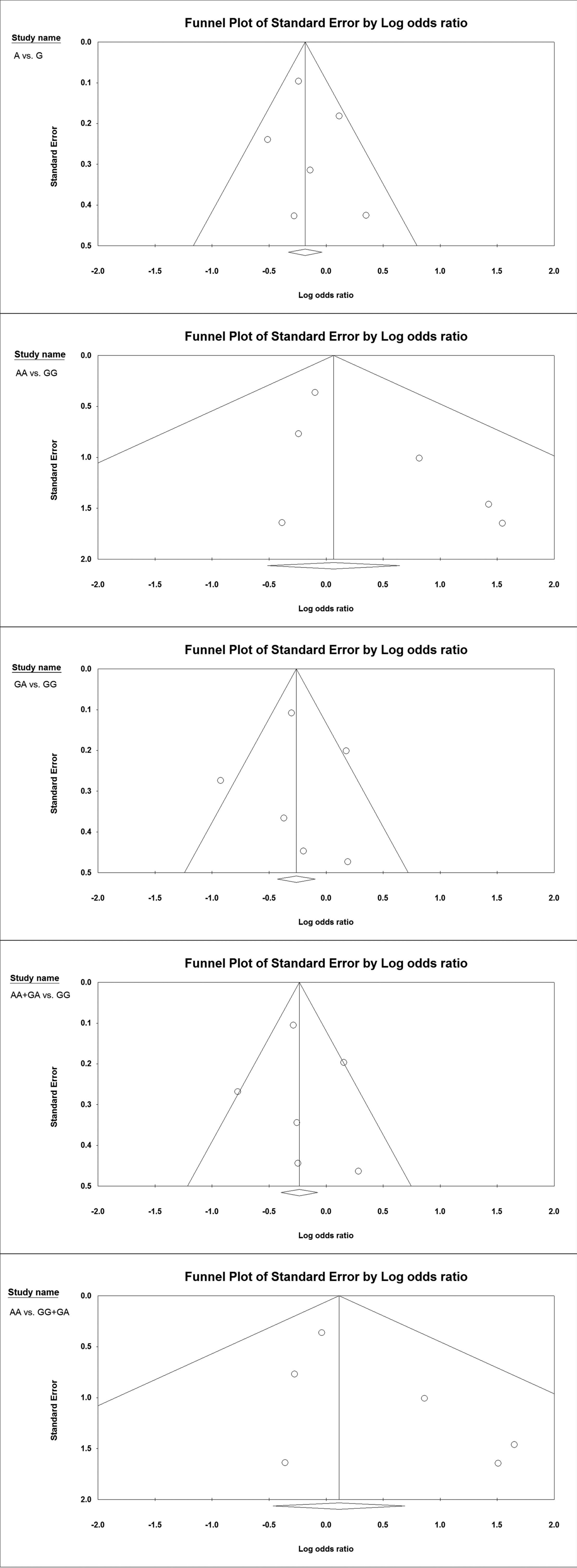
Funnel Plot of Standard Error by Log odds ratio
Study name
A vs. G
Standard Error
0.0
0.1
0.2
0.3
0.4
0.5
-2.0
-1.5
-1.0
-0.5
0.0
0.5
1.0
1.5
2.0
Log odds ratio
Funnel Plot of Standard Error by Log odds ratio
Study name
AA vs. GG
Standard Error
0.0
0.5
1.0
1.5
2.0
-2.0
-1.5
-1.0
-0.5
0.0
0.5
1.0
1.5
2.0
Log odds ratio
Funnel Plot of Standard Error by Log odds ratio
Study name
GA vs. GG
Standard Error
0.0
0.1
0.2
0.3
0.4
0.5
-2.0
-1.5
-1.0
-0.5
0.0
0.5
1.0
1.5
2.0
Log odds ratio
Funnel Plot of Standard Error by Log odds ratio
Study name
AA+GA vs. GG
Standard Error
0.0
0.1
0.2
0.3
0.4
0.5
-2.0
-1.5
-1.0
-0.5
0.0
0.5
1.0
1.5
2.0
Log odds ratio
Funnel Plot of Standard Error by Log odds ratio
Study name
AA vs. GG+GA
Standard Error
0.0
0.5
1.0
1.5
2.0
-2.0
-1.5
-1.0
-0.5
0.0
0.5
1.0
1.5
2.0
Log odds ratio

Study name | **Statistics with study removed** | **Odds ratio (95% CI) with study removed**

A vs. G

Study name	Point	Lower limit	Upper limit	Z-Value	p-Value
Oliveira et al. 2016	0.782	0.665	0.919	-2.990	0.003
Silva et al. 2015	0.829	0.713	0.965	-2.422	0.015
Felix et al. 2012	0.834	0.718	0.969	-2.380	0.017
Lima et al. 2012	0.817	0.704	0.949	-2.645	0.008
Cardoso et al. 2011	0.911	0.720	1.153	-0.774	0.439
Santos et al. 2002	0.862	0.738	1.006	-1.884	0.060
	0.832	0.718	0.963	-2.458	0.015

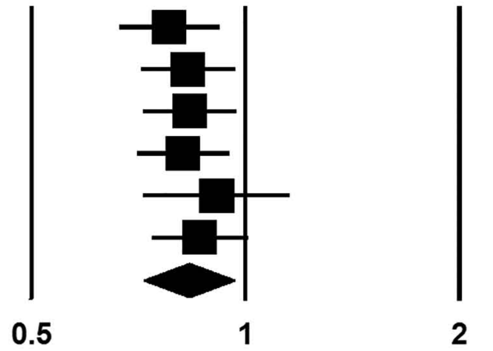

Study name | **Statistics with study removed** | **Odds ratio (95% CI) with study removed**

AA vs. GG

Study name	Point	Lower limit	Upper limit	Z-Value	p-Value
Oliveira et al. 2016	1.125	0.602	2.105	0.370	0.712
Silva et al. 2015	0.995	0.543	1.821	-0.018	0.986
Felix et al. 2012	1.083	0.602	1.950	0.267	0.790
Lima et al. 2012	1.016	0.565	1.829	0.053	0.958
Cardoso et al. 2011	1.466	0.542	3.964	0.754	0.451
Santos et al. 2002	1.007	0.558	1.818	0.024	0.981
	1.067	0.599	1.903	0.220	0.826

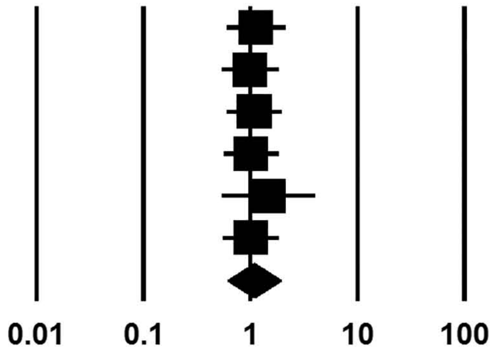

Study name | **Statistics with study removed** | **Odds ratio (95% CI) with study removed**

GA vs. GG

Study name	Point	Lower limit	Upper limit	Z-Value	p-Value
Oliveira et al. 2016	0.680	0.502	0.921	-2.487	0.013
Silva et al. 2015	0.786	0.539	1.146	-1.252	0.211
Felix et al. 2012	0.767	0.532	1.107	-1.416	0.157
Lima et al. 2012	0.738	0.520	1.047	-1.703	0.089
Cardoso et al. 2011	0.785	0.482	1.279	-0.971	0.331
Santos et al. 2002	0.866	0.677	1.107	-1.150	0.250
	0.772	0.559	1.068	-1.561	0.119

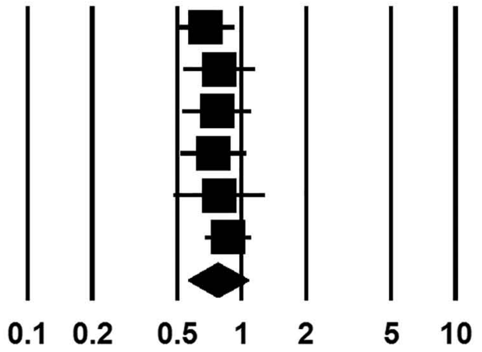

Study name | **Statistics with study removed** | **Odds ratio (95% CI) with study removed**

AA + GA vs. GG

Study name	Point	Lower limit	Upper limit	Z-Value	p-Value
Oliveira et al. 2016	0.728	0.610	0.869	-3.513	0.000
Silva et al. 2015	0.791	0.671	0.934	-2.768	0.006
Felix et al. 2012	0.791	0.671	0.931	-2.811	0.005
Lima et al. 2012	0.777	0.660	0.915	-3.022	0.003
Cardoso et al. 2011	0.858	0.663	1.110	-1.164	0.245
Santos et al. 2002	0.836	0.706	0.990	-2.082	0.037
	0.790	0.673	0.928	-2.865	0.004

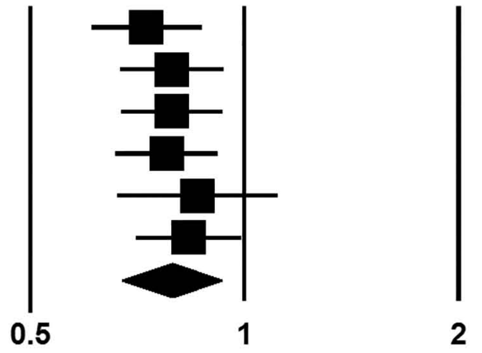

Study name | **Statistics with study removed** | **Odds ratio (95% CI) with study removed**

AA vs. GG+GA

Study name	Point	Lower limit	Upper limit	Z-Value	p-Value
Oliveira et al. 2016	1.197	0.641	2.236	0.563	0.573
Silva et al. 2015	1.044	0.571	1.909	0.138	0.890
Felix et al. 2012	1.137	0.632	2.045	0.429	0.668
Lima et al. 2012	1.069	0.594	1.922	0.222	0.824
Cardoso et al. 2011	1.501	0.556	4.052	0.801	0.423
Santos et al. 2002	1.049	0.582	1.891	0.158	0.874
	1.119	0.628	1.994	0.383	0.702

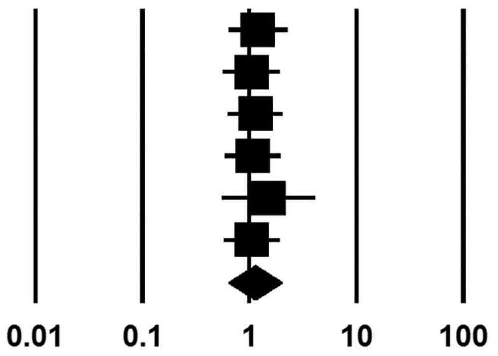

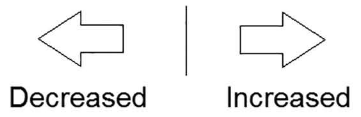

Supplement: Supplementary file 1 [file BSR20170806_Supp1.pdf]
